# Coccidioidomycosis among Workers Constructing Solar Power Farms, California, USA, 2011–2014

**DOI:** 10.3201/eid2111.150129

**Published:** 2015-11

**Authors:** Jason A. Wilken, Gail Sondermeyer, Dennis Shusterman, Jennifer McNary, Duc J. Vugia, Ann McDowell, Penny Borenstein, Debra Gilliss, Benedict Ancock, Janice Prudhomme, Deborah Gold, Gayle C. Windham, Lauren Lee, Barbara L. Materna

**Affiliations:** Centers for Disease Control and Prevention, Atlanta, Georgia, USA (J.A. Wilken);; California Department of Public Health, Richmond, California, USA (J.A. Wilken, G. Sondermeyer, D. Shusterman, J. McNary, D.J. Vugia, D. Gilliss, B. Ancock, G.C. Windham, L. Lee, B.L. Materna);; County of San Luis Obispo Public Health Department, San Luis Obispo, California, USA (A. McDowell, P. Borenstein);; California Department of Industrial Relations, Oakland, California, USA (J. Prudhomme, D. Gold)

**Keywords:** coccidioidomycosis, *Coccidioides*, occupational health, solar power farms, disease transmission, infectious, fungi, construction workers, California

## Abstract

Coccidioidomycosis is associated with soil-disruptive work in *Coccidioides*-endemic areas of the southwestern United States. Among 3,572 workers constructing 2 solar power–generating facilities in San Luis Obispo County, California, USA, we identified 44 patients with symptom onset during October 2011–April 2014 (attack rate 1.2 cases/100 workers). Of these 44 patients, 20 resided in California outside San Luis Obispo County and 10 resided in another state; 9 were hospitalized (median 3 days), 34 missed work (median 22 days), and 2 had disseminated disease. Of the 25 patients who frequently performed soil-disruptive work, 6 reported frequent use of respiratory protection. As solar farm construction in *Coccidioides*-endemic areas increases, additional workers will probably be exposed and infected unless awareness is emphasized and effective exposure reduction measures implemented, including limiting dust generation and providing respiratory protection. Medical providers, including those in non–*Coccidioides*-endemic areas, should suspect coccidioidomycosis in workers with compatible illness and report cases to their local health department.

## Abstract 2

In *Coccidioides*-endemic areas, effective exposure-reduction measures are needed.

Coccidioidomycosis and Solar Power Farms

Solar energy generation is an expanding industry, particularly in the southwestern United States (*1*). According to the experience in eastern San Luis Obispo County, California, construction of large-scale solar power–generating facilities (solar farms) in low-population rural areas may often require recruiting skilled laborers from outside the county or state. In California, multiple solar farms are being planned and constructed. During February 2013, after coccidioidomycosis was confirmed in several workers, the California Department of Public Health (CDPH) and the County of San Luis Obispo Public Health Department (SLOPHD) investigated illnesses among workers constructing 2 solar farms (A and B) in the county. We report the results of that investigation.

Coccidioidomycosis (Valley fever) is an infectious disease acquired by inhalation of soil-dwelling *Coccidioides* fungus spores, which are endemic to the southwestern United States, including San Luis Obispo County. After an incubation period of ≈1–3 weeks, ≈40% of infected persons experience an influenza-like illness, which can include cough, difficulty breathing, fever, and fatigue. Fewer than 5% experience disseminated disease, including meningitis, osteomyelitis, or cutaneous lesions (*2*). Severe or disseminated coccidioidomycosis requires treatment and can be fatal.

In recent years, reported cases of coccidioidomycosis and hospitalizations in California increased dramatically, peaking in 2011, particularly in the *Coccidioides*-endemic counties of the southern San Joaquin Valley (*3*–*7*). Outbreaks, however, have been reported infrequently and are usually associated with soil disruption (*8*–*15*). During December 2012–February 2013, an outbreak was suspected when SLOPHD notified CDPH of coccidioidomycosis diagnosed for 3 workers who had been constructing solar farm B and when 9 additional workers who had become ill were identified as having a potential connection to solar farms A or B. These additional workers were identified from the CDPH statewide coccidioidomycosis surveillance system and by occupational injury and illness reports on the basis of limited occupational data comments in the records. We investigated the extent of the outbreak, obtained clinical and epidemiologic data, identified work and environmental factors potentially contributing to exposures, and recommended preventive measures. The California Health and Human Services Agency’s Committee for the Protection of Human Subjects determined that this investigation was public health practice (i.e., not research).

## Methods

### Case Definition

A case was defined as laboratory-confirmed coccidioidomycosis in an employee of either solar farm A or B, with illness onset >1 week after beginning work but <1 month after the last workday at either solar farm. Criteria for laboratory-confirmed coccidioidomycosis were based on the 2011 Council of State and Territorial Epidemiologists and Centers for Disease Control and Prevention surveillance case definition for coccidioidomycosis (*16*). Criteria included 1) clinical presentation with influenza-like signs and symptoms, pneumonia, meningitis, or involvement of bones, joints, or skin; and 2) laboratory confirmation with cultural, histopathologic, or molecular evidence of *Coccidioides*, or positive serologic test results for coccidioidal IgM (by immunodiffusion, enzyme immunoassay [EIA], latex agglutination, or tube precipitin) or for coccidioidal IgG (by immunodiffusion, EIA or complement fixation).

### Case Finding

During December 2012–July 2014, we identified cases by using multiple sources. In California, health care providers and laboratories are required to report coccidioidomycosis diagnoses to the local health jurisdiction where the patient resides (i.e., reported as their permanent residence). Local health jurisdictions subsequently report cases to CDPH. Additionally, health care providers in California who evaluate an employee with a possible work-associated medical condition are required to complete a Doctor’s First Report of Occupational Injury or Illness (DFR) (*17*). We reviewed reports of coccidioidomycosis and DFRs to identify cases of coccidioidomycosis possibly associated with either solar farm. Additionally, we reviewed employee rosters and employer logs of injury and illness, mandated by the California Department of Industrial Relations, Division of Occupational Safety and Health (Cal/OSHA), from both solar farms’ contractors and subcontractors to match names with those reported to CDPH by local health jurisdictions and to a statewide database of workers’ compensation claims. We also sent a nationwide email alert to state and local health departments requesting information about coccidioidomycosis possibly acquired among workers constructing solar farms in San Luis Obispo County. Last, we sent a letter to all identified employer-designated health care providers and all health care providers who had evaluated or provided treatment for solar farm employees with a diagnosis of coccidioidomycosis to request that they report any solar farm employees with suspected coccidioidomycosis.

### Epidemiologic Investigation

During March 5–6, 2013, Cal/OSHA led a multiagency site visit at the solar farms to interview employers and employees, identify employer-referred health care providers, review employer injury and illness logs, observe work practices, identify potential exposure routes, and observe protective measures used. On the basis of that information, we developed a standardized telephone patient questionnaire for collection of data regarding demographics, work practices, worksite characteristics, patient clinical presentation and outcome, and preexisting medical conditions. For permanent residence, we defined the California *Coccidioides*-endemic counties as Fresno, Kern, Kings, Madera, San Luis Obispo, and Tulare and the less *Coccidioides–*endemic counties as all others, as previously described (*7*). We defined other states with possible coccidioidomycosis endemicity as Arizona, Nevada, New Mexico, Texas, and Utah. We also asked about dust exposure during the 4 weeks before disease onset, including work activities, dust exposure at or away from the worksite, and individual and worksite dust control and safety measures. From published literature (*18*), we identified manual digging, working in a ditch or trench, and operating heavy machinery as probable soil-disruptive exposures at solar farms. If patients were able to provide only an estimate for date or duration of these activities, we used the range midpoint for analyses; if patients were unable to provide an estimate, or if the date of symptom onset reported by the patient was later than the date of diagnosis, we ascertained this information from medical or employer records when possible. Patients’ estimates of frequency of performing soil-disruptive duties and high dust levels at the worksite were categorized as frequently (every day and once a week) or infrequently (rarely and never); respirator use frequency and hygienic practices were categorized as frequently (always and often) or infrequently (sometimes, rarely, and never).

We obtained employee rosters from as many employers as possible and determined the number of workers at each site. We received information about first and last day worked on rosters from 2 predominant employers at solar farm A (employers A1 and A2); using these rosters, we estimated employee time at risk for *Coccidioides* infection. Employee time at risk was defined as the number of days from the first to the last day worked onsite. Incidence among employees was compared with the incidence rate for San Luis Obispo County in 2012, which was calculated per 100,000 population (*19*). The number of incident cases among employer A1 and A2 employees was also compared with the average worker count by month by using the Spearman rank test. Statistical analyses were conducted by using SAS version 9.3 (SAS Institute, Inc., Cary, NC, USA), and p values <0.05 were considered significant.

## Results

**Figure 1:**
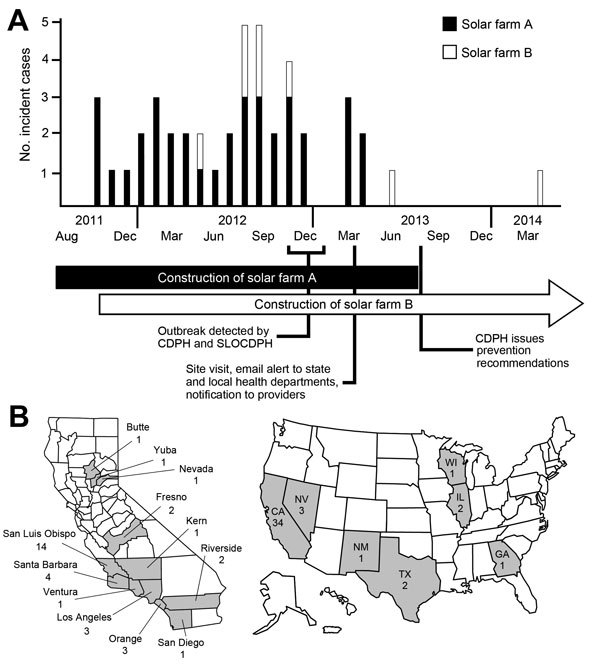
Distribution of outbreak cases of coccidioidomycosis among solar farm workers, by month of symptom onset and patients’ residence, San Luis Obispo County, California, USA, October 2011–April 2014. A) Number of cases listed by month of onset and by solar farm. Investigation timeline is displayed below the x-axis. B) Number of cases during this outbreak listed by patient-reported California county or state of permanent residence (gray shading). CDPH, California Department of Public Health; SLOPHD, County of San Luis Obispo Public Health Department.

**Figure 2:**
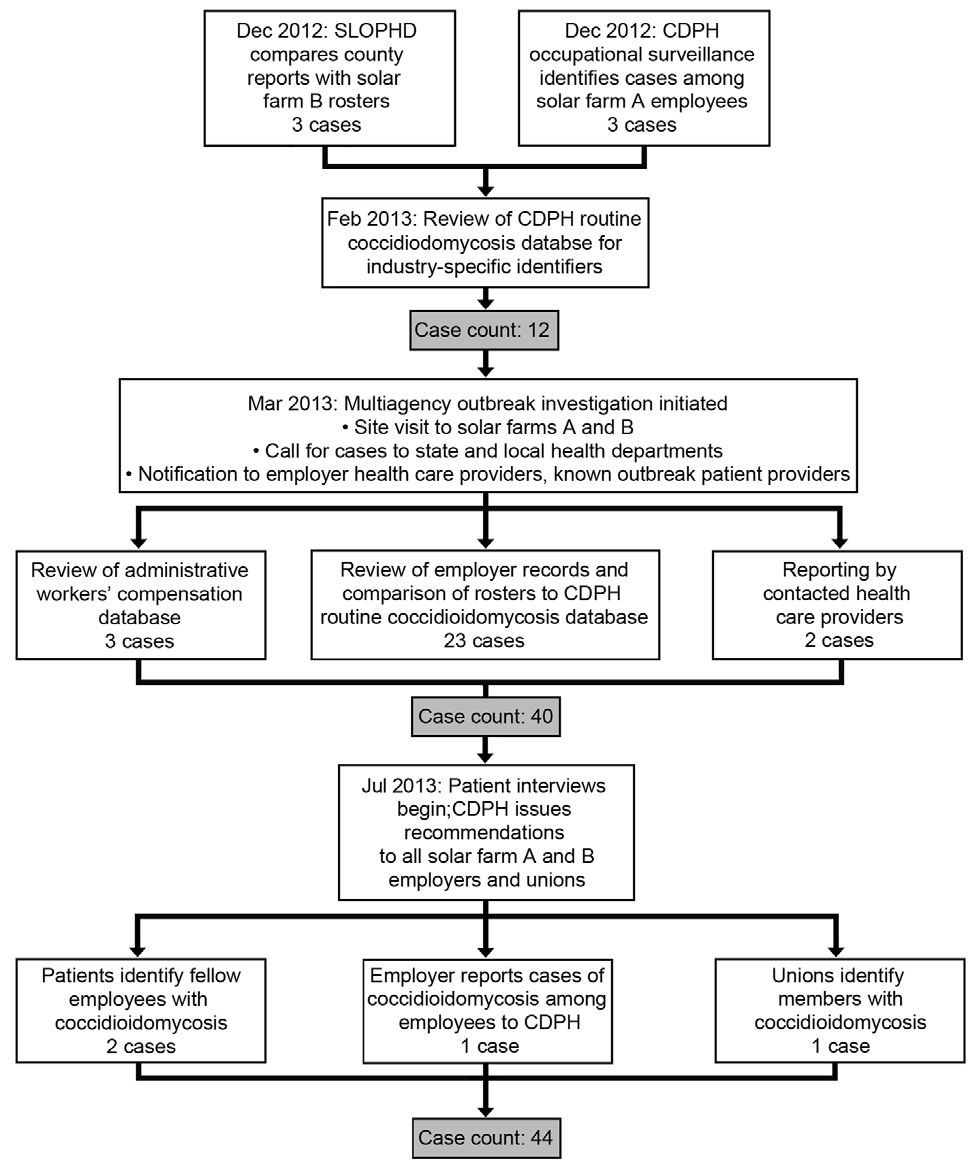
Flowchart of outbreak investigation of coccidioidomycosis among solar farm workers, San Luis Obispo County, California, USA, October 2011–April 2014. CDPH, California Department of Public Health; SLOPHD, County of San Luis Obispo Public Health Department.

Among 3,572 known employees at the 2 solar farms, we identified 44 patients with illness onset during October 4, 2011–April 15, 2014 (attack rate 1.2 cases/100 workers) (Figure 1, panel A). Cases were identified from multiple sources (Figure 2). Most were identified after the site visit, by review of employer records and comparison of rosters to CDPH coccidioidomycosis reports. Other cases were reported to CDPH by health care providers, by an employer, or by a union, or were identified by interviewed patients. Of note, no cases had been reported by employers to Cal/OSHA, SLOPHD, or CDPH before the site visit.

Of the 44 patients, 43 were interviewed; 41 completed the interview, and some refused or were unable to answer specific questions. Patients were interviewed a median of 335 (range 77–621) days after symptom onset. A total of 41 (93%) of the 44 patients were men; patients’ median age was 48 years. Of the 41 patients who completed the interview, 11 (26%) were Hispanic and 28 (68%) were white (Table 1). Varying job titles were reported by 43 patients; the most common titles reported were electrician (33%) and heavy equipment operator (26%).

**Table 1:** Demographic characteristics of 44 coccidioidomycosis patients who worked at 2 solar power–generating facilities, San Luis Obispo County, California, USA, with symptom onset October 2011–April 2014*

Characteristic	Value
Male sex, no. (%)	41 (93)
Median age, y (range)	48 (21–63)
Ethnicity, no. (%), n = 41	
Hispanic	11 (26)
Not Hispanic	28 (68)
Don’t know or data missing	2 (5)
Race, no. (%), n = 41	
White	28 (68)
Not white†	6 (15)
Don’t know or declined to state	7 (17)
Job title, no. (%), n = 43	
Electrician, lineman, or wireman	14 (33)
Heavy equipment operator	11 (26)
Laborer	6 (14)
Carpenter, ironworker, millwright, or mechanic	5 (12)
Manager or superintendent	4 (9)
Other	3 (7)
Permanent residence, no. (%)	
California, San Luis Obispo County	14 (32)
California, other *Coccidioides*-endemic county‡	3 (7)
California, less *Coccidioides–*endemic county	17 (39)
Other state with possible *Coccidioides* endemicity§	6 (14)
Any other state	4 (9)

*Of the 44 patients, 43 were interviewed and 41 completed the interview.  †Including African American, Filipino, Samoan, Native American, and multiracial. ‡Other *Coccidioides*-endemic counties are Fresno, Kern, Kings, Madera, and Tulare.  §Other possibly *Coccidioides*–endemic states are Arizona, Nevada, New Mexico, Texas, and Utah.

Of the 44 patients, 17 (39%) had a permanent address in the *Coccidioides*-endemic counties of California (14 [32%] in San Luis Obispo County), 17 (39%) in the less *Coccidioides*–endemic counties, 6 (14%) in another state with possible *Coccidioides* endemicity, and 4 (9%) in a non–*Coccidioides*-endemic state (Table 1; Figure 1, panel B). All interviewed patients with a permanent address outside of California reported a temporary address in San Luis Obispo County. Of the 20 interviewed California residents with permanent residence outside of San Luis Obispo County, 11 (55%) reported a temporary address: 8 in San Luis Obispo County, 2 in Kern County, and 1 unknown. Of the 3,572 identified workers, 39% resided in a California county other than San Luis Obispo and 21% resided outside California.

Patients’ clinical characteristics at time of illness are displayed in Table 2. Of the 44 patients, 17 (39%) visited an emergency department at some point during their illness and 9 (20%) were hospitalized for 2–17 (median 3) days. For 2 (5%) patients, the diagnosis was disseminated disease: 1 to skin and 1 to bone. The median time from first day of work to symptom onset was 105 (range 10–638) days; 10 (23%) patients reported symptom onset <2 months after beginning work. Of the 41 patients who provided symptom data, >80% reported fatigue, night sweats, weakness, difficulty breathing, fever, cough, joint/muscle pain, and weight loss.

**Table 2:** Clinical characteristics of 44 coccidioidomycosis patients who worked at 2 solar power–generating facilities, San Luis Obispo County, California, USA, with symptom onset October 2011–April 2014*

Characteristic	Value
Visited emergency department, no. (%)	17 (39)
Hospitalized, no. (%)	9 (20)
Length of hospitalization, median no. days (range)	3 (2–17)
Disseminated disease, no. (%)	2 (5)
No. days to symptoms from first day at worksite, median (range), n = 43	105 (10–638)
Symptoms, no. (%), n = 41	
Fatigue	41(100)
Night sweats	39 (95)
Weakness	38 (93)
Difficulty breathing	37 (90)
Fever	35 (85)
Cough	33 (80)
Joint or muscle pain	33 (80)
Weight loss	33 (80)
Chest pain	29 (71)
Headache	29 (71)
Rash or other skin lesions	24 (59)
Missed work, no. (%), n = 41	34 (83)
Days missed work, median no. (range), n = 34	22 (1–547)
Diagnosed following first visit to provider, no. (%), n = 41	9 (22)
No. days to laboratory diagnosis from symptom onset, median (range), n = 43	23 (5–267)
Frequency of positive laboratory result by diagnostic method, no. (%)	
Complement fixation, immunodiffusion, or tube precipitin	37 (84)
Complement fixation only	10 (23)
Immunodiffusion only	10 (23)
Tube precipitin only	2 (5)
Immunohistochemistry or culture only	3 (7)
IgG/IgM enzyme immunoassay only	4 (9)

*Of the 44 patients, 43 were interviewed and 41 completed the interview.

A limited number of interviewed patients reported chronic medical conditions (excluding hypertension) before symptom onset. None reported corticosteroid use, and only 1 reported an immunocompromising condition (cancer, diagnosed after coccidioidomycosis onset).

Of the 41 patients who completed the interview, 34 (83%) reported missing work because of illness (median 22 days); 2 had missed work for >18 months at the time of interview. We estimate that a minimum of 9.1 person-years of work were lost because of illness. A total of 11 (27%) patient reported that as of the interview date they were still experiencing health effects that interfered with work or other physical activity.

Only 9 (22%) of 41 patients reported having received a diagnosis of coccidioidomycosis after their first symptom-related visit to a health care provider. The median time from symptom onset to laboratory diagnosis was 23 (range 5–267) days. Of the 44 patients, 37 (84%) received a diagnosis by using >1 of the following serologic tests for *Coccidioides* exposure: complement fixation, immunodiffusion, or tube precipitin. Of the remaining 7 patients, 4 received a diagnosis by serologic IgG or IgM EIA results only and 3 by only immunohistochemical test or *Coccidioides* culture results.

### Site and Work Characteristics

**Figure 3:**
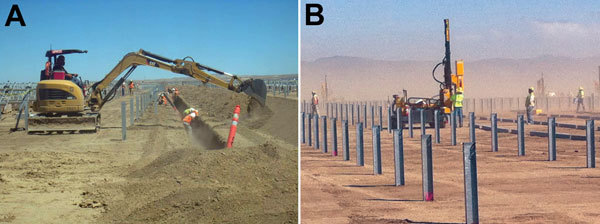
Conditions during solar farm construction in San Luis Obispo County, California, USA. A) Localized dust generation associated with a soil-disruptive activity. Photograph was taken during the week of July 28–August 3, 2013 (courtesy of Aspen Environmental Group). B) Ambient dust exposure because of high-wind conditions. Photo was taken on March 5, 2013 (courtesy of Dennis Shusterman).

Both solar farms are located near the southwestern side of San Joaquin Valley, ≈10 miles apart in sparsely populated, arid grassland where it is frequently windy and dusty. Construction of solar farm A occurred during September 2011–October 2013 and of solar farm B during November 2011–December 2014 (Figure 1, panel A). Both solar farms encompass thousands of acres. Installation of solar photovoltaic panel arrays involved leveling the ground, digging trenches for installation of underground electric cables, performing electrical work in trenches, driving piles to install panel rigs, and ancillary digging manually or by machine. Employees can be exposed to dust by wind or by being in close proximity to soil-disruptive activities (Figure 3). The San Luis Obispo County permit approval for solar farm projects requires minimizing exposure to dust and providing coccidioidomycosis safety training to all employees (*20*,*21*). It further requires monitoring for visual opacity caused by suspended dust and shutting down work if the opacity exceeds 20%.

The 43 interviewed employees reported working outdoors a median of 10 (range 0–11) hours/day. A total of 25 (58%) reported frequently performing soil-disruptive exposure activities, including digging, working in a ditch or trench, or operating heavy machinery (Table 3); of these 25, only 6 (24%) reported frequently using respiratory protection. Of 42 patients, 39 (93%) reported substantial dust levels at the worksite most days. Of 41 patients, most (31 [76%]) reported frequently driving home in work clothes, potentially increasing exposure to *Coccidioides*. None reported exposure to dust outside of work, and only 3 (7%) reported working at a construction site other than the solar farms <4 weeks before symptom onset.

**Table 3:** Potential worksite *Coccidioides* exposures and use of control measures during 4 weeks before symptom onset by workers at 2 solar power–generating facilities, San Luis Obispo County, California, USA, October 2011–April 2014*

Exposure	No. (%)		
Frequently	Infrequently	Don’t know
Work duties involved, n = 43†			
Manual digging	12 (28)	31 (72)	0
Working in a ditch or trench	19 (44)	24 (56)	0
Operating heavy machinery‡	8 (19)	35 (81)	0
Any of the above	25 (58)	18 (42)	0
High dust levels, n = 42†	39 (93)	0	3 (7)
Used a respirator, n = 41§¶	10 (24)	31 (76)	0
At the end of work, did you†			
Drive home in work clothes, n = 41	31 (76)	9 (22)	1 (2)
Wash up before leaving work, n = 39	19 (49)	20 (51)	0
Shower immediately after getting home, n = 41	34 (83)	5 (12)	2 (5)

*Of the 44 patients, 43 were interviewed and 41 completed the interview. Some patients did not answer certain questions. †Frequently includes the responses of “every day” and “once a week”; infrequently includes the responses of “rarely” and “never.” ‡One of 8 worked with heavy equipment with an enclosed cab and high-efficiency particulate air (HEPA)–filtered air-conditioning every day. §Frequently includes the responses of “always” and “often”; infrequently includes the responses of “sometimes,” “rarely,” and “never.”  ¶Does not include 1 employee who reported operating heavy equipment with an enclosed cab and HEPA-filtered air-conditioning every day.

Of the 44 patients, 36 (82%) were employees at solar farm A (Figure 1, panel A); of these, 29 worked for employer A1, 3 worked for employer A2, and 4 worked for other employers. The number of incident cases by month for employers A1 and A2 combined was associated with the average worker count onsite (Spearman ρ = 0.62; p<0.01), indicating that individual workers’ temporal risk for infection was relatively constant. The incidence rate among employees of employers A1 and A2 combined was 5,168 cases/100,000 person-years, 135 times (95% CI 84–215 times) the 2012 incidence rate of 38.4 cases/100,000 person-years for San Luis Obispo County (*19*) and 109 times (95% CI 82–145 times) the 2012 incidence rate of 47.4 cases/100,000 person-years for adult men in San Luis Obispo County (A. McDowell, unpub. data). We were unable to compare incidence rates among employers and across worksites because we were unable to obtain rosters that included first and last day of work from employers with coccidioidomycosis-diagnosed employees, other than employers A1 and A2.

Of 41 patients, 36 (88%) reported having received safety training regarding Valley fever; however their descriptions of the training provided ranged widely, from comprehensive safety training that addressed how to minimize dust exposure to more limited notification (required by San Luis Obispo County, condition of the permit) about the risk for Valley fever, symptom recognition, and diagnosis. Although not specifically asked during the interview, 8 patients volunteered that when seeking care for symptoms, they had asked their health care provider to test for coccidioidomycosis.

### Public Health Response

On July 22, 2013, CDPH provided interim recommendations for preventing and reporting coccidioidomycosis among workers to all identified employers at both solar farms and associated unions based, in part, on the CDPH published guidelines (*22*). The recommendations extended beyond the original (local) permitting requirements, and included the following:

- Improve worksite dust-control measures, including stabilizing disturbed soil areas, increasing soil watering frequency, using soil binders, and covering excavated soil.
- Equip all earth-moving equipment and trucks with high-efficiency particulate air ([HEPA]-filtered) enclosed cabs to protect the operator.
- Implement and enforce criteria for suspending work on the basis of wind and dust conditions.
- Provide all outdoor workers access to National Institute for Occupational Safety and Health–approved respiratory protection (respirators with particulate filters designated N95, N100, P100, or HEPA) within the context of a comprehensive, OSHA-compliant respirator program including provision of medical clearance, fit testing, and training (*23*). (Respirator use was recommended when conducting or in close proximity to soil-disturbing work, and for exposure to excessive wind-blown dust, but not necessarily for the entire work shift in recognition of possible variation in exposure levels and the potential risk of heat stress.)
- Provide clean coveralls daily to employees; encourage workers to remove coveralls and work shoes before entering vehicles to leave the worksite.
- Improve compliance by employers and their designated health care providers with reporting cases to local health jurisdictions, workers’ compensation carriers, and Cal/OSHA.

Solar farm B, which continued construction after solar farm A was completed, reported implementing the CDPH recommendations and having reduced the amount of grading. During September 2013, multiple employers at both solar farms were cited by Cal/OSHA for failure to implement appropriate control measures for coccidioidomycosis as part of their injury and illness prevention programs, failure to provide adequate employee respiratory protection, and failure to report serious employee illnesses and hospitalizations resulting from coccidioidomycosis (*24*). Despite the general contractors’ awareness of *Coccidioides* in the soil and implementation of some dust-reduction steps, the measures used were insufficient to prevent an excess of laboratory-confirmed cases over expected rates, including illnesses among patients who reported infrequently engaging in soil-disruptive work. Therefore, more effective methods to control dust and better adherence to exposure-control standards were deemed necessary.

## Discussion

This outbreak of coccidioidomycosis among workers in the growing solar power industry is concerning because additional solar farms are being planned for *Coccidioides*-endemic areas. Large-scale construction, including solar farm construction, might involve substantial soil disturbance for months, and many employees, particularly from non–*Coccidioides*-endemic areas, probably lack immunity to *Coccidioides*.

Given the frequently dusty conditions, as well as the fact that coccidioidomycosis can be a mild and self-limiting illness for which persons do not seek medical attention, we believe that additional employees at these 2 solar farms were probably exposed to *Coccidioides* and had undiagnosed illness of mild or moderate severity. Thus, the number of confirmed cases is probably an underestimate of the extent of the outbreak. Even so, the health consequences, medical resources required, and lost productivity for the identified patients have been substantial.

Although some patients may have been exposed to *Coccidioides* spores away from the worksite, no patients reported exposure to dust outside of work. Furthermore, the high incidence rate among these workers relative to background rates demonstrates that occupational exposure played a predominant role in disease incidence. Because nearly half of the patients reported infrequent or no soil-disruptive work, it is likely that factors other than direct work activities contributed to coccidioidomycosis risk on this worksite. Dust-producing activities and high winds in this area expose workers to ambient dust from inside and outside the work area (Figure 3, panel B); therefore, employers should assess whether respirators (in addition to wetting the local soil and other dust control measures) are necessary for reducing dust exposure for outdoor workers. Respiratory protection may be particularly advisable in the near field, such as work in trenches where other dust-reduction techniques may be less effective. Admittedly, no published study has compared incidence among 2 similarly exposed groups to address the effectiveness of air-purifying respirators for preventing coccidioidomycosis, and wearing an N95 filtering facepiece or half-face elastomeric respirator for a prolonged period is uncomfortable. However, because air-purifying respirators greatly reduce exposure to airborne particles (*25*,*26*), outdoor workers in *Coccidioides*-endemic areas should be protected from exposure to a recognized hazard and provided this personal protective equipment to use during soil-disturbing activities or when outdoors during dust storm or high winds. Also, employers should be required by local permitting agencies to prepare (and adhere to) comprehensive coccidioidomycosis-prevention strategies, including plans for minimizing dust generation, limiting workers’ exposure, and establishing criteria for stopping work when wind or dust is excessive or uncontrollable. Prompt reporting of employee illnesses to local health jurisdictions and to OSHA programs will help identify worksites where control measures need improvement.

A previous study determined that patients in Arizona who were aware of coccidioidomycosis received a diagnosis earlier than those who were unaware (median 20 vs. 25 days) (*27*). The relatively rapid diagnoses of illnesses during the outbreak described in this article demonstrates the value of training workers to recognize and report their illness to supervisors and for employers to promptly refer ill employees to health care providers. Health care providers in non–C*occidioides*-endemic areas might not consider coccidioidomycosis in the differential diagnosis of prolonged febrile respiratory illnesses. Reports such as this one can help increase the index of suspicion for this condition and prompt providers to ask patients about potential environmental dust exposures acquired elsewhere.

Describing the occupational burden of coccidioidomycosis is challenging because existing data sources are not comprehensive. Although incidence rates estimated by using statewide workers’ compensation claims have been reported (*18*), these estimates include only those employees who sought workers’ compensation; in the outbreak described in this article, few cases were initially identified by review of workers’ compensation claims. Similarly, in a 2012 outbreak among outdoor workers, only 2 of 10 cases were initially identified by review of DFRs (*15*). The variety of data sources necessary to describe the scope of this outbreak highlights the need for improved recognition and reporting of occupational illness by medical providers to public health jurisdictions as required under public health regulations and, in instances of serious illness involving hospitalization, for employer compliance with mandated reporting to occupational safety and health regulatory agencies.

This investigation had limitations. The calculated relative risk for workers, although impressive, compares denominators of total person-time worked at the solar farm for workers and assumes a stable population for San Luis Obispo County. We were unable to obtain rosters from all employers and unable to estimate person-years of work time for all employees at the 2 solar farms. As a consequence, we recommend that in the future, conditions for permit approval for large construction projects in *Coccidioides*-endemic areas should require that complete employee rosters including date of birth be provided to the local health jurisdiction. Case-finding was also complicated because a substantial number of employees resided outside San Luis Obispo County and California. In addition, interviews were conducted a median of 335 days after symptom onset, giving rise to the potential for recall bias.

In conclusion, unless awareness is emphasized and effective prevention measures are implemented, additional construction in *Coccidioides*-endemic areas, including solar power facility construction, will probably expose workers to *Coccidioides*, thus leading to additional infections. Awareness and prevention of coccidioidomycosis among all personnel at these and other similar construction sites should be included among the priorities for employee safety. Medical providers should consider work-related coccidioidomycosis when evaluating construction workers with prolonged febrile respiratory illness, particularly after work in central or southern California or in Arizona, and medical providers should follow all statutory requirements for documenting and reporting occupational illness.
